# Editorial: Transcriptional regulation and posttranslational modifications in plant growth and development under abiotic stresses

**DOI:** 10.3389/fpls.2024.1454335

**Published:** 2024-08-07

**Authors:** Ali Movahedi, Saeid Kadkhodaei, Liming Yang

**Affiliations:** ^1^ State Key Laboratory of Tree Genetics and Breeding, School of Life Sciences, Nanjing Forestry University, Nanjing, China; ^2^ Agricultural Biotechnology Research Institute of Iran, Agricultural Research, Education and Extension Organization, Isfahan, Iran

**Keywords:** abiotic stress, transcriptional regulation, posttranslational modifications, gene expression, stress adaptation and resistance

Plants are constantly exposed to various abiotic stresses, causing complex regulatory mechanisms for adaptation and survival. This editorial investigates the intricate molecular processes that underpin plant responses to these stresses, specifically transcriptional regulation and posttranslational modifications (PTMs). Understanding how plants control gene expression and protein activity in response to abiotic stress is critical for increasing crop resilience and productivity. Transcriptional regulation refers to controlling gene expression through transcription factors, whereas PTMs (such as phosphorylation, acetylation, ubiquitination, sumoylation, etc.) can influence protein function and stability. Researchers can identify potential targets for genetic manipulation to improve plant stress tolerance by understanding the complex interplay of these regulatory mechanisms.

## Transcriptional regulation in response to abiotic stresses

Transcriptional regulation, or the control of gene expression at the transcription level, is critical for plant responses to environmental stimuli. Two major studies on the role of transcriptional regulation in plant stress responses are discussed. Previous research has examined the development of different leaf shapes in *Populus euphratica* during drought (Xu et al.). Transcriptomic analysis revealed significant changes in the expression of genes involved in hormone signaling, stress response, and leaf development. This study demonstrated the plasticity of plant development in response to environmental challenges, specifically how plants can reprogram their transcriptome to adapt to water scarcity. The formation of heteromorphic leaves is an excellent adaptation strategy, allowing plants to maximize water use efficiency during drought conditions. Key genes involved in this process were identified, providing important insights into the molecular mechanisms that underpin drought tolerance in woody plants.

## Phosphate starvation in Arabidopsis

An investigation has already been conducted comparing the functions of PHR1, PHL1, and PHL4 transcription factors in Arabidopsis under phosphate starvation (Wang et al.). Their study discovered how these transcription factors have overlapping and distinct functions in regulating phosphate levels. This research highlighted the intricate nature of transcriptional networks that play a role in adapting to nutrient stress. The differential roles of closely related transcription factors were highlighted, demonstrating the fine-tuning capabilities of plant regulatory systems. Understanding these intricate networks can develop strategies to enhance crop resilience to nutrient-poor conditions.

## Salicylic acid-induced flowering in duckweed

Insights into posttranscriptional regulation during salicylic acid-induced flowering in duckweed (*Lemna gibba*) were already provided (Fu et al.). Discrepancies between mRNA and protein levels were discovered after combining transcriptome and proteome analyses, indicating extensive posttranscriptional control. This research highlighted the significance of considering more than changes in gene expression to comprehend how plants respond fully. The differences in mRNA and protein levels indicate that posttranscriptional mechanisms are important in regulating developmental transitions under stressful conditions. Such findings emphasized the importance of integrative approaches when studying plant stress responses.

## Mechanisms of posttranscriptional regulation

Posttranscriptional regulation involves a variety of mechanisms, such as alternative splicing, mRNA stability, and RNA-mediated gene silencing. The roles of these mechanisms in plant adaptation to abiotic stresses were reviewed by Floris et al. (2009). Alternative splicing has been shown to produce multiple protein isoforms from a single gene, potentially increasing the functional diversity of the proteome under stress. Rapid changes in transcript levels were made possible by mRNA stability regulation, which allowed for quick responses to environmental changes. RNA-mediated gene silencing, including microRNA-mediated regulation, provided additional control over gene expression during stress responses. This section delves into posttranslational modifications (PTMs), which are critical for dynamically regulating protein function in response to environmental stressors.

## Role of ubiquitination and sumoylation

The roles of ubiquitination and sumoylation in plant stress responses were highlighted by Guerra et al. (2015). The activity and stability of key regulatory proteins were modified by these PTMs, ensuring appropriate gene expression patterns during stress conditions. Rapid changes in protein levels in response to stress were often targeted by ubiquitination for degradation. Protein function, localization, or interactions were altered by sumoylation. The interplay between these modifications provided a sophisticated system for fine-tuning cellular responses to abiotic stresses.

## Arginine methylation in AGO proteins

The role of arginine methylation in ARGONAUTE (AGO) proteins was explored by Martín-Merchán et al. (2024). The functionality of AGO proteins is affected by symmetric arginine dimethylation of their N-terminal extensions, which leads to changes in small RNA loading and protein interactions. This research underscored the significance of PTMs in regulating gene expression at the posttranscriptional level. Small RNA-mediated gene silencing was central to AGO proteins, and their modification through arginine methylation added another layer of regulation to this process. Such discoveries shed light on the elaborate mechanisms through which plants regulate their gene expression in reaction to environmental cues.

## *PeHVA22* gene family in passion fruit

The *PeHVA22* gene family in passion fruit (*Passiflora edulis*) was already characterized (Hou et al.). The diverse roles of *PeHVA22* genes in various abiotic stress responses were demonstrated through expression profiling analysis. This study highlighted the importance of gene family expansion and functional diversification in plant stress adaptation. The complex roles of this gene family in stress responses were uncovered by examining the expression patterns of multiple gene family members across different stress conditions. The multifaceted nature of plant stress responses was revealed by the *PeHVA22* gene family study, exemplifying how integrative approaches can provide a comprehensive view of plant adaptation to abiotic stresses. Combining genomic analysis with expression profiling provided a holistic view of how this gene family contributes to stress adaptation in passion fruit.

## Conclusion

This Research Topic highlighted the complex relationship between transcriptional regulation and posttranslational modifications in plant abiotic stress responses. Integrating multiple omics approaches gave us a holistic view of regulatory networks, improving our understanding of plant biology. The studies in this editorial showed the complexity of plant stress responses, from transcriptional reprogramming to posttranslational protein modifications. Plants use a variety of regulatory mechanisms to adapt to environmental changes. Understanding these molecular mechanisms is crucial as climate change threatens global agriculture. These studies improve our understanding of plant biology and may lead to more resilient crop varieties that can thrive in harsh environments.

## Author contributions

AM: Conceptualization, Project administration, Supervision, Validation, Writing – original draft. SK: Conceptualization, Validation, Writing – review & editing. LY: Conceptualization, Funding acquisition, Project administration, Supervision, Validation, Writing – review & editing.
